# Memory bias in the temporal bisection point

**DOI:** 10.3389/fnint.2015.00044

**Published:** 2015-07-07

**Authors:** Joshua M. Levy, Vijay M. K. Namboodiri, Marshall G. Hussain Shuler

**Affiliations:** Department of Neuroscience, Johns Hopkins UniversityBaltimore, MD, USA

**Keywords:** memory, bias, temporal bisection, interval production, interval timing, timing and time perception

## Abstract

The ability to time intervals confers organisms, including humans, with many remarkable capabilities. A common method for studying interval timing is classification, in which a subject must indicate whether a given probe duration is nearer a previously learned short or long reference interval. This task is designed to reveal the probe duration that is equally likely to be labeled as short or long, known as the temporal bisection point. Studies have found that this bisection point is influenced by a variety of factors including the ratio of the target intervals, the spacing of the probe durations, the modalities of the stimuli, the attentional load, and the inter-trial duration. While several of these factors are thought to be mediated by memory effects, the prototypical classification task affords no opportunity to measure these memory effects directly. Here, we present a novel bisection task, termed the “Bisection by Classification and Production” (BiCaP) task, in which classification trials are interleaved with trials in which subjects must produce either the short or long referents or their midpoint. Using this method, we found a significant correlation between the means of the remembered referents and the bisection points for both classification and production trials. We then cross-validated the bisection points for production and classification trials by showing that they were not statistically differentiable. In addition to these population-level effects, we found within-subject evidence for co-variation across a session between the production bisection points and the means of the remembered referents. Finally, by using two sets of referent durations, we showed that only memory bias-corrected measures were consistent with a previously reported effect in which the ratio of the referents affects the location of the bisection point. These results suggest that memory effects should be considered in temporal tasks.

## Introduction

Organisms rely on a wide range of temporal information to guide their behavior (Buhusi and Meck, 2005). At one end of the spectrum, organisms require sub-second information to guide movement (Edwards et al., 2002; Schirmer, 2004). At the other end, their circadian rhythms are entrained by temporal cycles that span days (Czeisler et al., 1999). In between these extremes, organisms must be able to evaluate the length of temporal intervals on the order of seconds to hours to guide their decision-making (Richelle and Lejeune, 1980; Gallistel, 1990; Kacelnik and Brunner, 2002; Sohn and Carlson, 2003). Because of the importance of this type of temporal information, interval timing has been studied extensively in laboratory settings.

A common method for studying interval timing in humans is a classification task (Allan and Gibbon, 1991; Wearden, 1991). While the precise form of this task has been modified many times, its essential component is that subjects are required to classify sample temporal intervals as short or long. Typically, this classification relies on the subject remembering previously learned short and long reference intervals (i.e., similarity method). The obtained data is the percentage that the subject chooses to label an interval “long” as a function of probe duration length. From this data, the bisection point, or probe duration at which a subject is equally likely to choose “short” or “long,” can be inferred.

Several theories of timing and time perception make predictions about the location of the bisection point. One of the most influential theories of timing, scalar expectancy theory (Gibbon, 1977), posits that the bisection point lies at the geometric mean of the short and long reference intervals (Allan and Gibbon, 1991). Another timing theory, which assumes a difference rule for comparing a probe duration to the short and long referents, predicts that the bisection point lies at the arithmetic mean of the referents (Wearden, 1991). More contemporary theories have been brought to bear in an attempt to systematically explain the observed variations in the bisection point location from the harmonic mean to the arithmetic mean (Killeen et al., 1997; Kopec and Brody, 2010) and rationalize such variations in terms of reward-rate maximization (Namboodiri et al., 2014) and optimal temporal risk assessment (Balci et al., 2011; Coskun et al., 2015). Given that these many theoretical accounts of timing make specific predictions about the location of the bisection point, obtaining accurate and meaningful measurements of the bisection point is crucial.

Indeed, many factors have been shown to affect the location of the bisection point. These factors include the ratio of the target intervals (Wearden and Ferrara, 1996; Allan, 2002b), the spacing of the probe durations (Wearden and Ferrara, 1995; Brown et al., 2005), the modalities of the stimuli (Penney et al., 1998, 2000; Penney and Tourret, 2005; Cheng et al., 2008, 2011), the attentional load (Fortin et al., 1993; Fortin and Breton, 1995; Jones and Wearden, 2004; Gil and Droit-Volet, 2011), and the inter-trial duration (Spetch, 1987; Lieving et al., 2006). Several of these factors are thought to affect memory and, thereby, the subjective representation of time. Increasing the cognitive load, for example, by requiring subjects to observe emotionally charged faces can cause either an overestimation or underestimation of time on a temporal bisection task, depending on whether the stimulus is arousing (e.g., angry face) or attention-demanding (e.g., shameful face), respectively (Gil and Droit-Volet, 2011). Analogously, requiring subjects to engage in tasks which demand working memory, like remembering a series of digits, will cause distortions in temporal production (Fortin et al., 1993; Fortin and Breton, 1995). Similarly, varying inter-trial duration is believed to affect the degree of memory trace degradation and, thereby, whether a subject is more likely to label an interval “short” (choose-short effect) or “long” (choose-long effect; Spetch, 1987; Lieving et al., 2006).

It is not surprising that these factors affect time perception given that memory is a key component of several interval timing models. In pacemaker accumulator models, the number of pulses generated by a pacemaker during the reference interval is stored in memory and compared against the number of pulses generated by a probe duration (Treisman, 1963). This comparison serves as a proxy for comparing the length of a probe duration to the referent. Thus, affecting the rate of the pacemaker during the referent/probe duration, or altering values stored in memory, can affect the subjective representation of time. Pharmacological manipulations showing a dissociation of the effects on memory from effects on perception have bolstered this view (Meck, 1983, 1996). Another model of interval timing, the striatal beat-frequency model, also reserves a key role for memory (Matell and Meck, 2000, 2004). In this model, striatal neurons compare ongoing cortical oscillatory patterns to prior patterns of activation that co-occurred with the expiration of an interval. Still other models have posited that the strength of a memory trace is itself an internal clock (Staddon and Higa, 1999; Staddon, 2005) which naturally meshes with the notion that alterations in memory affect time perception.

Given the importance of memory in temporal perception, several variants of the prototypical bisection task have been developed to study its role. One variant of the similarity method, in which subjects are explicitly taught to recognize the short and long reference intervals, is the partition method, in which subjects must infer the range of intervals and, concomitantly, what constitutes a short and long interval (Wearden and Ferrara, 1995, 1996; Droit-Volet and Rattat, 2007). Interestingly, the results from these experiments indicate that both methods yield similar outcomes, suggesting that subjects do not compare probe durations directly to referents. Instead, it is believed that subjects translate these referent durations into a mental representation threshold above which a probe duration is classified as long and below which a probe duration is classified as short, a process which may be integral in cross-domain comparisons of quantities (Balci and Gallistel, 2006). Another variant of the prototypical task in which referents are provided at the beginning of the session only (i.e., “no-referents” method), is the “fixed-referents” task, in which the same referents are displayed before each trial (Allan and Gerhardt, 2001). By replaying the reference intervals before each trial, the contribution of memory to the decision process should be reduced. Yet another variant, the “roving-referents” task (Rodríguez-Gironés and Kacelnik, 1995, 1998) changes the referent intervals on a trial-by-trial basis, thereby reducing or eliminating the effect of memory.

Entirely different methods have also been employed to study temporal perception. One of the most prevalently used methods is a reproduction task in which subjects observe a temporal interval on each trial and must reproduce it as accurately as possible (Jazayeri and Shadlen, 2010; Cicchini et al., 2012). In this way, the translation of real time into subjective time can be observed across a wide range of time points while minimizing the role of memory. Another method requires subjects to periodically produce taps at a frequency set by a guiding stimulus (Merchant et al., 2008). In this method, the readout is the temporal error in tapping after the guide stimulus has been removed. Yet another production method asks subjects to wait for a time specified by a verbal cue (e.g., 4 s). While relevant to some applications, this method does not fully control for subjects’ prior experience and also may be confounded by the interaction between magnitude (e.g., numerosity) and temporal perception (Dormal and Pesenti, 2007; Xuan et al., 2007; Oliveri et al., 2008). Though informative, these experimental methods do not, by construction, reserve a large role for memory and are, therefore, not ideal for studying its effects.

Our goal was to develop a method for directly assessing how memory affects the bisection point location in prototypical bisection tasks. To do this, we developed the “Bisection by Classification and Production” (BiCaP) task which interleaves trials in which subjects must use their memory to produce the short or long reference intervals or their midpoint with trials in which they must classify probe durations as short or long. Both classification and production trials yield an estimate of the bisection point (the point at which the subject classifies the interval “long” 50% of the time and the produced midpoint interval, respectively). We anticipated that the bisection point generated from each method would not be differentiable. Further, we hypothesized that the biases in the memory of the referents, as measured on a fraction of production trials, would co-vary with the location of the bisection point for both classification and production trials. This is indeed what we found. Additionally, the BiCaP task is a novel and powerful method for studying temporal perception generally.

## Materials and Methods

### Subjects

Twenty healthy, human subjects aged 22–38 participated in this study. All subjects were recruited from The Johns Hopkins University, were naïve about the purpose of the study and gave consent to participate. All procedures were approved by the Institutional Review Board (IRB).

### Apparatus

Subjects were placed in a quiet room in front of a MacBook Pro. Instructions were displayed on the screen and simultaneously read aloud by the experimenter. All responses were registered by clicks on a wireless mouse. For production trials, the subjects had to left-click to start and stop the interval. For classification trials, the subjects had to left- or right-click to classify the interval as short or long, respectively. Clicks prior to the end of the interval were not registered. To proceed to a subsequent trial, the subject was required to tap the space bar once. The trial type was indicated at the top of the screen. Stimuli were presented and responses were collected by custom-made code written in Java (JDK 6.0_65).

### Task

#### Training

Prior to testing, subjects experienced a training phase that consisted of three parts. In the first part, *observation*, subjects were instructed to observe short and long intervals. Each interval was labeled as either “SHORT” or “LONG” at the top of the screen. 12 observation trials were given. In the second part, *classification training*, subjects were shown an unlabeled short or long reference interval and instructed to classify it. Depending on whether they classified it correctly or incorrectly, they were shown a smiley or frowny face for 1 s. In order to pass classification training, the subject had to correctly classify three short and three long intervals consecutively by type (with the trial types interleaved with one another). In the third part, *production training*, subjects were instructed to produce the short or long reference interval by instructions at the top of the screen. Depending on whether the response was close enough (i.e., within a window centered on the appropriate interval whose half-width was a Weber fraction of 0.2), they were shown a smiley or frowny face for 1 s. Additionally, subjects received feedback about how far from the appropriate interval they were off (in milliseconds, rounded to the nearest integer). This feedback would appear on the left or right side of the screen depending on whether the response was short or long, respectively. The text was in green font for correct responses and red for incorrect responses.

Production training itself was divided into three stages, in which the subject produced short referents only, long referents only, and then short and long referents inter-mixed. For training on the short trials only, the mean ± the standard deviation of the subject’s last 8 responses needed to be between a lower (LB_short_) and upper (UB_short_) bound (in seconds). The same calculation was performed for the long trials except the range was LB_long_ to UB_long_. During the final part, in which trials were inter-mixed, these criteria were applied to short and long trials separately.

Subjects were placed into one of two groups. In one group, the 1v5 group, the subjects (10) were trained with a short referent that was 1 s in length and a long referent that was 5 s in length. For production training in this group, LB_short_ = 0.6 s, UB_short_ = 1.4 s, LB_long_ = 3.75 s, and UB_long_ = 6.25 s. In other words, the mean ± the standard deviation on short production trials had to be between 0.6 and 1.4 s over the last eight responses, whereas the mean ± the standard deviation on long production trials had to be between 3.75 and 6.25 s. In the other group, the 2v4 group, subjects (10) were trained with a short referent that was 2 s and a long referent that was 4 s. For production training in this group, LB_short_ = 1.5 s, UB_short_ = 2.5 s, LB_long_ = 3 s, and UB_long_ = 5 s. The subjects were never explicitly told which group they were in nor what the length of the referent stimuli were.

#### Experimental Testing

After passing training, subjects were told they would be asked to “(1) CLASSIFY the sample interval (based on whether it’s closer to the short or long target interval) or (2) PRODUCE the short interval, long interval or MIDPOINT between them” during testing. All subjects performed a novel task called the “Bisection by Classification and Production” (BiCaP). This task combines a prototypical classification task (Allan and Gibbon, 1991; Wearden, 1991) with novel aspects. For the classification component, subjects were required to classify a probe duration as either short or long (by left- or right-clicking, respectively). The novel aspect of the task, the production component, required subjects to produce the short referent, the long referent, or the midpoint between them (by left-clicking to start and stop the interval). Note that while subjects produced the long and short referents during training, they were required to produce a third interval, the midpoint, during testing. Trial types were indicated at the top of the screen. For classification trials, the instruction was “CLASSIFY” whereas for the production trials the instruction was “PRODUCE SHORT,” “PRODUCE LONG,” or “PRODUCE MIDPOINT.” No feedback was provided during testing.

### Stimuli

The stimuli were blue ovals (122 × 73 pixels) that appeared at the center of a white background (1220 × 730 pixels). The duration that the blue interval appeared on the screen constituted the length of the interval. On classification trials, the subject could left or right-click after the stimulus had ended. On production trials, a blue oval would appear upon clicking to start the interval and would disappear upon clicking to end the interval.

During training, only short and long referents were displayed. On each trial, the probability of receiving a short or long referent (or being asked to produce the short or long referent in the third stage of production training) was equal. During testing, many probe durations were displayed. For the 2v4 group, the probe durations used were {2.0, 2.33, 2.66, 3.0, 3.33, 3.66, 4.0} and for the 1v5 group, the probe durations were {1.00, 1.67, 2.33, 3.00, 3.67, 4.33, 5.00} (i.e., seven linearly spaced intervals between the short and long referent, inclusive). In all, 110 trials were performed by each subject during testing. A block structure was used in which each block consisted of 11 trials and there were 10 blocks per session. A block consisted of seven classification trials (one of each probe duration), two midpoint production trials, and two referent production trials (one short and one long). Within a block, the presentation order was random.

### Analysis Methods

All analysis was performed using custom scripts written in MATLAB, The Mathworks Inc.

#### Analysis of Classification:

To analyze classification data, we numerically fit a psychometric function for the probability that an interval *t* is labeled as closer to “long” by the generalized logistic function shown below

$$p(\text{"}long")=\frac{p_{3}-p_{4}}{1+e^{-\frac{\left(t-p_{2}\right)}{p_{1}}}}+p_{4}$$

where *p_1_, p_2_, p_3,_* and *p_4_* represent free-fit parameters. Once the best fit parameters were obtained using non-linear regression, the bisection point [the duration for which *p(“long”) = 0.5*] was calculated as

$$Bis=p_{2}-p_{1}\log\left(\frac{p_{3}-0.5}{0.5-p_{4}}\right)$$

The Weber fraction was measured as the difference limen, which is defined as half the difference in the durations corresponding to *p(“long”) = 0.75* and *p(“long”) = 0.25*, divided by the bisection point. This is shown below

$$Error=\frac{p_{1}}{2}\left(\log\left(\frac{p_{3}-0.25}{0.25-p_{4}}\cdot \frac{0.75-p_{4}}{p_{3}-0.75}\right)\right)$$

The sensory Weber fraction was calculated as the ratio between the error and bisection point.

In order to calculate the 95% confidence intervals, we used bootstrapping. This was performed for 1000 runs by randomly drawing different trials with replacement and calculating the resultant Weber fraction for each run. The 95% confidence intervals were measured from this sampling distribution.

To test whether the slopes of covariation between the bias-corrected arithmetic mean (i.e., the mean of the produced short and long referent intervals) with respect to production bisection point and classification bisection point were significantly different, we used bootstrapping. For each bootstrap, we randomly selected subjects with replacement and calculated the difference in these slopes. This procedure was repeated 2,000 times to obtain a two-tailed *p*-value for whether the sampling distribution of the difference in slopes was significant.

To look for within-subject evidence of memory bias in the production bisection point, we divided the data from each subject’s session into 10 equal blocks of 11 trials each (which was the experimentally imposed block length). For each block we averaged the two midpoint productions and computed the mean of the short and long referent productions. We then regressed the averaged midpoint against the mean of the referent productions per block and obtained the slope of regression for each subject. The mean of the slopes across subjects within the 1/5 s or 2/4 s cohort was compared to a null distribution of slopes from each cohort. The null distribution was created by randomly shuﬄing the data within a subject’s session, computing the slope of regression for each subject, and taking the average of the slopes from each subject. This procedure was repeated 1,000 times to obtain a two-tailed *p*-value for whether the average of the slopes across subjects within each cohort was significantly different from the mean of the null distribution.

## Results

**FIGURE 1:**
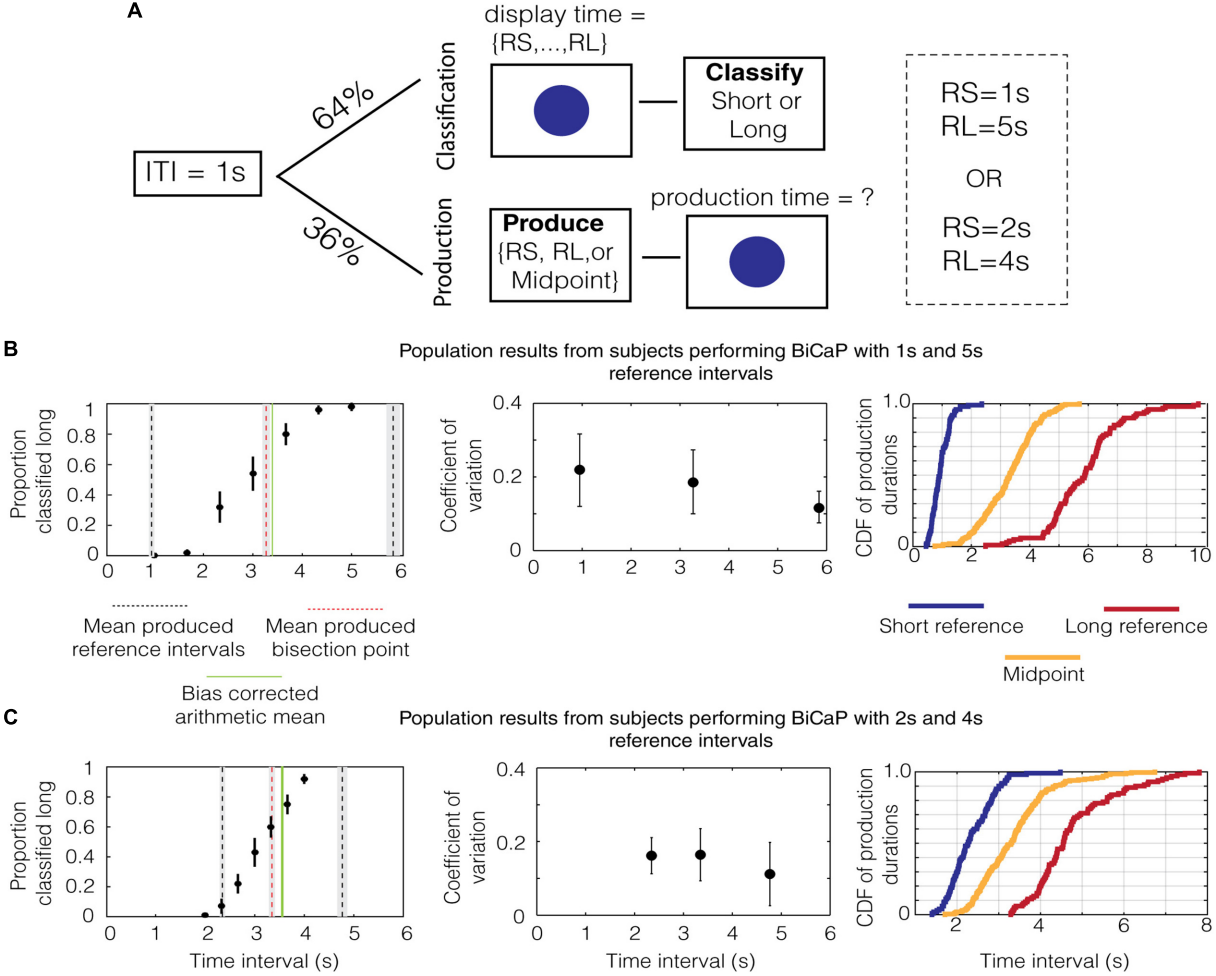
**Performance on the BiCaP Task. (A)** Design of the BiCaP task which consists of two trial types—classification and production—pseudo-randomly interleaved. **(B)** The proportion that a given probe duration is classified long (left panel, black dots with SEMs across subjects) for the population in the 1/5 s referent group. Mean produced intervals for the short reference (black dotted line), long reference (black dotted line), and midpoint (red dotted line) are shown along with the SEM (gray bars). The bias-corrected arithmetic mean (AM; i.e., the AM of the produced short and long referent intervals) is shown in green. Note that both the produced midpoint and the bias-corrected AM lie above the true AM of the 1/5 s reference intervals (i.e., 3 s). The mean coefficient of variation (CV) across subjects for each production interval is shown with SEMs on the middle panel. The empirical cumulative distribution function (CDF) of production times for the short reference (blue), long reference (red), and midpoint (yellow) is shown on the right panel. **(C)** The population data for the 2/4 s group.

To assess the effects of memory on the bisection point, we developed the “Bisection by Classification and Production” (BiCaP) task (**Figure 1A**), which consists of classification (top) and production (bottom) trials. Using this design, we were able to compare the bisection point produced from classification trials with the midpoint from production trials and, simultaneously, assess what the subjects believe the reference intervals to be. We used two sets of reference intervals, 1/5 and 2/4 s, for different cohorts.

We analyzed data from subjects in the 1/5 s group to generate descriptive statistics for classification and production trials. As expected, data on classification trials in this group showed a monotonically increasing relationship between the length of the probe duration and the proportion that the interval was classified “long” (**Figure 1B**, black dots with SEMs across subjects). After fitting a sigmoid function to the data (*R*^2^ = 0.99), we found that the interpolated point at which the subject was equally likely to classify a probe duration as short or long (i.e., the bisection point) was 2.859 s. This point has an associated Weber ratio, which is a measure of the standard deviation around a point divided by its mean. For classification, the Weber ratio is the difference limen, defined as half the difference in interval lengths at the points where the subject responded long 75% and 25% of the time, divided by the bisection point. The Weber ratio of the classification bisection point for the 1/5 group was 0.212.

Mean responses on production trials are shown in the same panel (**Figure 1B**, lines). The mean produced bisection point, shown in red, is 3.269 ± 0.066 s (SEM). The mean produced short and long interval, shown in black, is 0.948 ± 0.032 and 5.844 ± 0.128 s, respectively. Whereas the mean produced short interval was only slightly shorter than 1 s, the mean produced long interval was nearly a whole second above its true value of 5 s. Therefore, while the arithmetic mean of the referent intervals is 3 s, the bias-corrected arithmetic mean (i.e., the AM of the produced short and long reference intervals), shown by a solid green line, was 3.396 ± 0.066 s.

Next, we assessed whether the scalar property (Gibbon, 1977), which states that the standard deviation in time estimation grows linearly with temporal magnitude, holds for individual subjects in the 1/5 group. To do this, we calculated the Weber-like fraction (Weber, 1851), or coefficient of variation (i.e., standard deviation/mean), for production across the range of times tested (i.e., short reference, midpoint, and long reference). The median values for the population of subjects are plotted with the error bars indicating the interquartile range (**Figure 1B**, middle). To test whether Weber’s law holds across the pool of subjects, we compared the coefficient of variation (CV) for the population across the three intervals (short reference, production bisection point and long reference). We found a significant difference in median [*p* = 0.012, χ^2^(29) = 8.83, Kruskal–Wallis test] for produced intervals with the 1 and 5 s references. Thus, our data does not support Weber’s law in the production of these intervals. Such a decreasing trend for the CV has also been observed in humans previously, wherein shorter intervals have larger CVs than longer ones (Wearden and Lejeune, 2008).

These analyses were repeated for the data from the 2/4 group. As was the case for the 1/5 group, the proportion of probe durations classified “long” monotonically increased with the length of the probe duration (**Figure 1C**, black dots). The classification bisection point was 3.180 s and the Weber fraction was 0.155. As before, averaged responses on production trials are shown in the same panel (**Figure 1C**, lines). The mean production bisection point was 3.347 ± 0.061 s. The mean production time for the short and long interval was 2.347 ± 0.054 and 4.767 ± 0.101 s, respectively. Consequently, the bias-corrected arithmetic mean of the two referents is larger than three (3.557 ± 0.057 s). Repeating the same analysis with the Weber fractions for produced intervals in the 2/4 group, we found no difference in median across the range of intervals [*p* = 0.24, χ^2^(29) = 2.82, Kruskal–Wallis test], which is consistent with Weber’s law.

**FIGURE 2:**
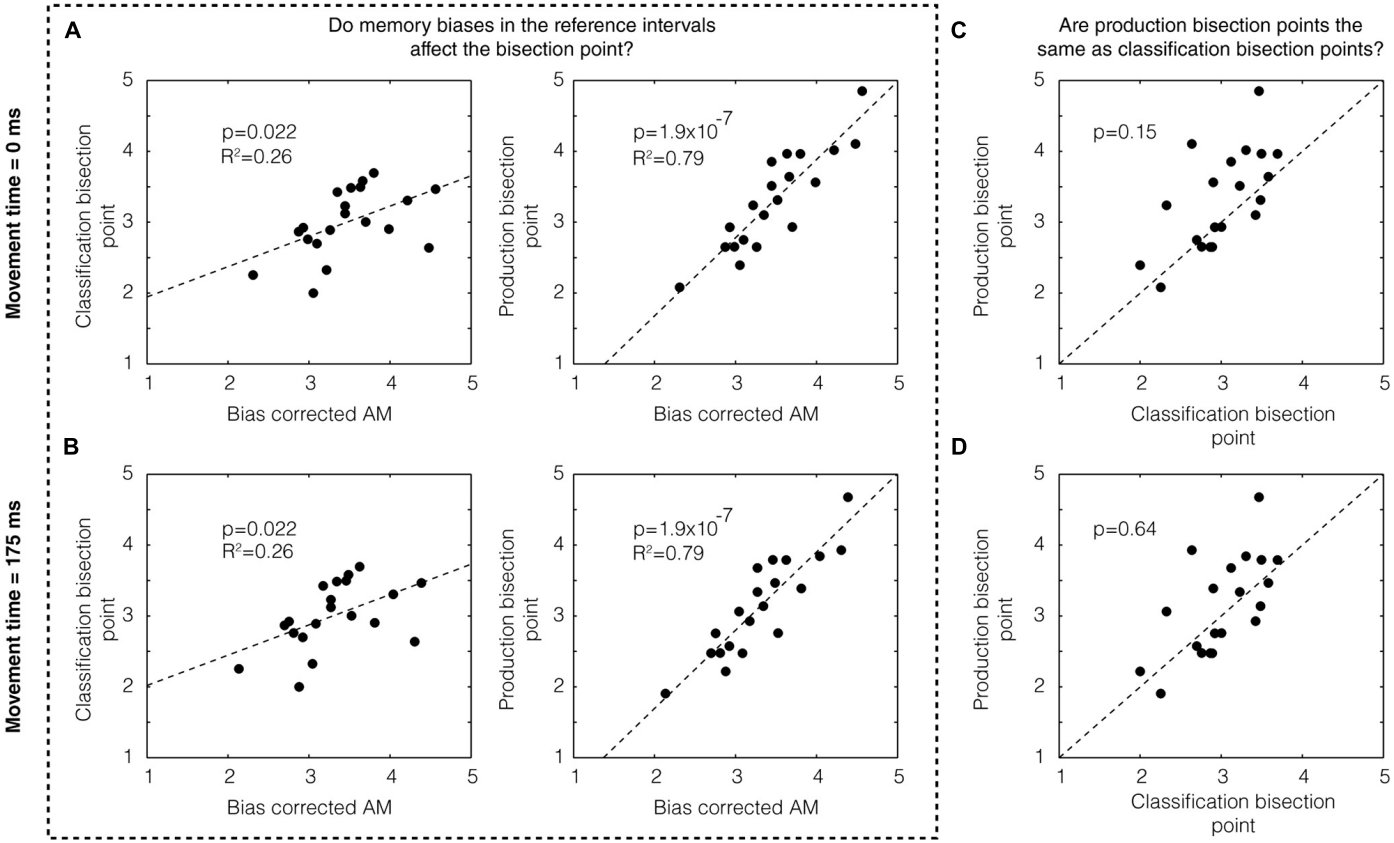
**Memory bias in production and classification. (A)** We found a statistically significant correlation between the bias-corrected AM and the classification bisection point (*p* = 0.022) assuming a movement time 0 ms (left panel). We also found a highly significant correlation (*p* = 1.9 × 10^-7^) with the bias-corrected AM for the production bisection point (right panel). **(B)** These results do not change assuming a different movement time (i.e., 175 ms). Production bisection points were not statistically distinguishable from classification bisection points **(C)** if movement time was assumed to be zero (*p* = 0.15, two-tailed Mann–Whitney *U*-test, *U*_18_ = 356, *z* = -1.45) or **(D)** if movement time was assumed to be 175 ms (*p* = 0.64, *U*_18_ = 392, *z* = -0.47).

Next, we sought to assess the degree to which memory biases may have affected the location of the bisection point on both classification and production trials. To this end, we examined the correlation between the bias-corrected mean and the bisection points across subjects for classification trials (*p* = 0.022, *R*^2^ = 0.26) and production trials (*p* = 1.9 × 10^-7^, *R*^2^ = 0.79) and found both to be significant (**Figure 2A**, top). (Separating by groups, we found: classification 1/5 s: *p* = 0.175, *R*^2^ = 0.22; production 1/5 s: *p* = 0.002, *R*^2^ = 0.70; classification 2/4 s: *p* = 0.098, *R*^2^ = 0.30, production 2/4 s: *p* = 3.14 × 10^-5^, *R*^2^ = 0.90.) We examined the co-variation between classification and production bisection points (**Figure 2C**) and also found no significant difference between these groups (*p* = 0.15, two-tailed Mann–Whitney *U*-test, *U*_18_ = 356, *z* = -1.45).

Whereas classification is a purely perceptual measure of the subjective estimate of time, production includes both a perceptual component and a motor component. If the movement time associated with the motor component is automatically compensated for by the brain, we could treat the movement time to be zero (as we have done above). If it is not, however, the movement time could simply be added to the end of the interval. Using a prior study in which the mean movement time of clicking a computer mouse was estimated to be approximately 175 ms (using EEG to detect the moment of movement initiation; Houlihan et al., 1998) as a guide, we subtracted this number from the subjects’ responses on production times. As expected, this manipulation did not affect the degree of correlation between the bias-corrected mean and the bisection points (**Figure 2B**). It did, however, make the difference between production and classification bisection points even weaker (*p* = 0.64, *U*_18_ = 392, *z* = -0.47; **Figure 2D**).

**FIGURE 3:**
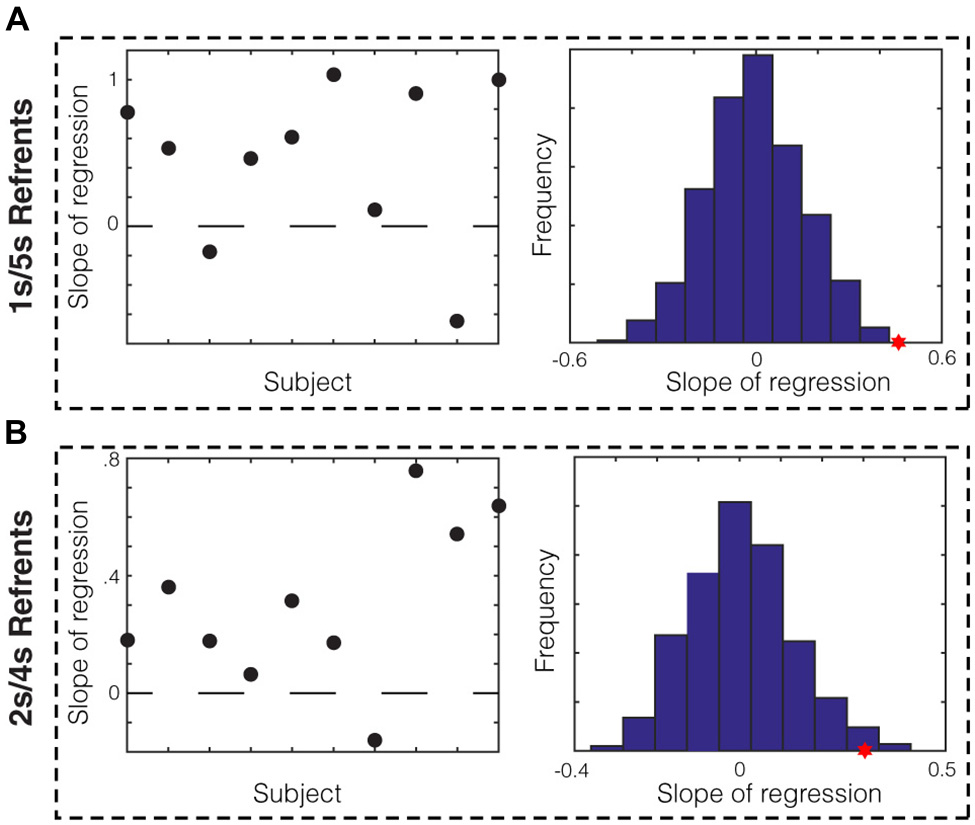
**Within-subject analysis of memory bias in production. (A)** Slope of regression in the 1/5 s referent group between the mean of the produced referents and the averaged midpoint production per block for each subject. Data from 8/10 subjects show positive slopes. The sampling distribution of slopes was calculated by bootstrapping (right). The average slope for the 1/5 s groups (red star) was significantly higher than the null distribution (*p* < 0.001). **(B)** This analysis was repeated for the 2/4 s referent group. Data from 9/10 subjects showed positive slopes and the average slope was significantly higher than that of the null distribution (*p* = 0.007).

We next sought to determine whether there was evidence for memory bias within data from individual subjects. To do this, we divided each subject’s session into ten equal blocks and asked whether the average midpoint production in a given block correlated with the mean of the produced short and long referent in that block. We found that for the 1/5 s group, data from eight out of ten subjects showed positive slopes of regression (**Figure 3A**, left). We compared the average slope across subjects (**Figure 3A**, red star) to that of the null distribution of slopes calculated by bootstrapping (see Materials and Methods) and found that it was significantly different (*p* < 0.001). We repeated this analysis for the 2/4 s group. We found that data from nine out of ten subjects in this group showed positive slopes (**Figure 3B**, left) and the average slope across subjects was, again, significantly higher that that expected by chance (**Figure 3B**, red star; *p* = 0.007). Therefore, we found evidence from individual subjects that memory bias in the production of the referents correlated with the produced bisection point across the session.

Prior work has shown that the ratio of referent intervals can affect the location of the bisection point (Wearden and Ferrara, 1996; Allan, 2002b). A meta-analysis of classification data (Kopec and Brody, 2010) reported that the bisection point lies near the geometric mean (GM) for ratios of 2 or less and lies near the arithmetic mean (AM) for ratios of 4 or greater. Since our referent interval sets spanned this range, we are able to assess whether our data follows this trend. We first performed these calculations for the population without taking into account memory bias. Neither the production nor the classification bisection points exhibited the referent ratio effect: the production bisection points for both groups were significantly greater than the arithmetic mean (1/5 s: *p* = 9.2 × 10^-5^, *W*_199_ = 6845, *z* = -3.91; 2/4 s: *p* = 1.16 × 10^-6^, *W*_199_ = 5995, *z* = -4.86, two-tailed Wilcoxon signed rank test against median = 3) and the classification bisection points were higher for the 2/4 group (3.180 s) than the 1/5 group (2.859 s) and were found to be significantly different by bootstrapping (*p* < 0.001).

Given that the bisection points correlate with a bias-corrected measure, we sought to determine whether the referent ratio effect could be observed for the remembered referents instead. To this end, we created a distance index defined as:

$$\text{Distance}\mathrm{index=}\frac{\mathrm{bisection}\mathrm{point}-\mathrm{bias}\mathrm{corrected}\mathrm{GM}}{\mathrm{bias}\mathrm{corrected}\mathrm{AM-}\mathrm{bias}\mathrm{corrected}\mathrm{GM}}$$

**FIGURE 4:**
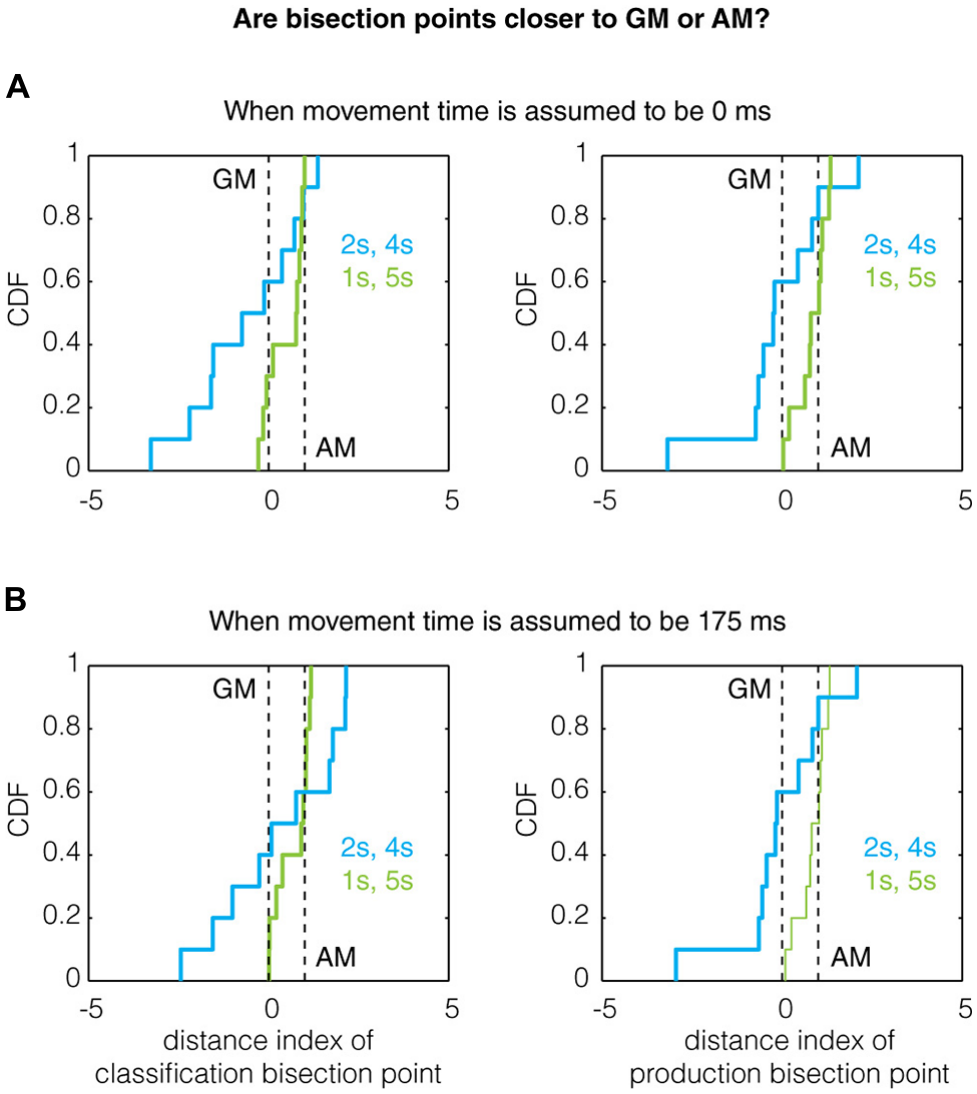
**The effect of referent ratio on the bisection point location.** Previous work has demonstrated that the bisection point transitions toward the arithmetic mean (AM) from the geometric mean (GM) as the ratio of the referents increases (Wearden and Ferrara, 1996; Allan, 2002b; Kopec and Brody, 2010). Here, we show the cumulative distribution function (CDF) of the distance indexes (defined in Results) for classification and production bisection points. Qualitatively, the data obeys this trend as the median bisection point across all subjects is closer to the AM for the 1/5 s group (referent ratio of 5) than the 2/4 s group (referent ratio of 2) for both classification (left) and production (right) trials assuming **(A)** a movement time of 0 ms and **(B)** a movement time of 175 ms. A quantitative treatment of this data can be found in Section “Results.”

Thus, when D.I = 0, the bisection point equals the bias-corrected GM and when D.I = 1, the bisection point equals the bias-corrected AM. Analyzing the data this way, we found evidence for the referent ratio effect in production trials (but not classification trials for which the power was likely too low). Qualitatively, the mean of the distance index for the 1/5 s group was higher than that of the 2/4 s group for both classification and production trials (**Figure 4A**), even when assuming a non-zero movement time (**Figure 4B**). Quantitatively, the production bisection point for the 1/5 s group was significantly different from the GM (Two-tailed Mann–Whitney *U*-test with *n* = 10, *p* = 0.0091, *U* = 140, *z* = 2.608; *n* = 10, *p* = 0.0046, *U* = 143, *z* = 2.8347, for 0 and 175 ms movement times, respectively) but not the AM (*p* = 1, *U* = 105, *z* = 0, for both movement times). While these comparisons were not significant for the 2/4 s group, the production bisection points for the 1/5 and 2/4 s groups were significantly different in closeness to the GM and AM (0.0376, *U* = 77, *z* = -2.0788 for both movement times), as expected by the referent ratio effect.

## Discussion

Considering the effect of memory bias on the bisection point location is critical as different theoretical accounts of timing make different predictions about where it lies (Allan, 2002b). Theories that hypothesize a linear mapping between real time and subjectively represented time, and assume a difference rule in comparing a probe duration to each of the referents, predict that the bisection point lies at the arithmetic mean (Wearden, 1991). Scalar timing theory, which favors a ratio rule for comparing a probe duration to each referent, predicts that the bisection point lies instead at the geometric mean (Allan and Gibbon, 1991). Still other theories, which perhaps best accord with the preponderance of the behavioral data, predict that the bisection point location will vary, based on task parameters and subjective statistics, all the way from the arithmetic mean down to the harmonic mean (Killeen et al., 1997; Kopec and Brody, 2010; Namboodiri et al., 2014). Given the importance of the bisection point location to these theories, it is critical to account for the sources of bias in measuring the bisection point.

We sought to measure the memory bias in bisection point location by employing both temporal classification and production methods. While several studies have examined the similarities and differences between temporal classification and production (Ivry and Hazeltine, 1995; Merchant et al., 2008), the “Bisection by Classification and Production” (BiCaP) task we used is unique in that these methods are employed simultaneously (by interleaving different trial types within a single session). Using this method, we were able to directly address whether memory biases can affect the location of the bisection point. Indeed, we found that the bias-corrected arithmetic mean, calculated from the mean produced time of the short and long referents, co-varied with the classification bisection point (**Figure 2**, column 1). We also found co-variation with the production bisection point (**Figure 2**, column 2). While we found significant co-variation between the bias-corrected arithmetic mean and the production bisection point within the 1/5 and 2/4 s groups separately, we did not observe the same within each group for the classification bisection point. Perhaps the bias-corrected arithmetic mean is not as predictive of the classification bisection point location as it is derived from production trials, and, consequently, higher power would be needed to reveal significance within these groups. We also found evidence for within-subject co-variation between the production bisection point and the mean of the remembered referents across the session (**Figure 3**). Taken together, we show that considering memory bias of the referents helps account for the variability in the bisection point observed on both classification and production trials. Such bias is difficult to directly measure in prototypical classification tasks in which only data about the bisection point (both its location and associated CV) is obtained.

An alternative explanation of our findings is that the observed biases are attributable to clock speed changes. This interpretation is challenged by the fact that we do not observe biases in production of the 1 s reference duration whereas we do observe a large bias for production of the 5 s duration. It is possible, however, that this differential observation is due to the fact that different mechanisms may underlie sub- and supra-second timing (Wittmann, 2009). To further address this question, we looked at whether a clock speed interpretation could apply to the 2/4 s group. If clock speed is accounting for the observed effects, then we might expect to find a change in produced times of the reference durations from training to testing. Assuming the clock speed changes were linear, we should expect to see a proportional change for the short reference duration as the long reference duration (which in this case, are 2 and 4 s, respectively). To test this, we performed a two-sided Wilcoxon signed rank test and found that the medians of the ratios for the 2 s duration (that is, the ratio of the production duration during testing to the duration during training) were significantly different from those of the 4 s duration (*p* = 0.0039). We repeated this for the 1/5 s group and, not surprisingly, found that the medians were again significantly different (*p* = 0.0059). This suggests that our effects are not attributable to linear variations in clock speed. It is difficult, of course, to rule out the possibility that some arbitrary non-linear clock speed variation may account for our effects, but a linear relationship in commonly assumed is pacemaker-accumulator models (Meck, 1983).

Importantly, prior work has studied the effects of reference memory and found no effect on time perception (Allan and Gerhardt, 2001; Wearden and Bray, 2001; Allan, 2002a). Allan and Gerhardt directly addressed this issue by comparing the prototypical bisection task to a “roving-referents” task (Rodríguez-Gironés and Kacelnik, 1995, 1998), in which new referents are shown prior to every trial and the subjects must determine whether a probe interval is more similar to the first or second referent. In this study, no difference was observed between the prototypical task and the roving referents version of the task. One explanation for this conflicting result is that this study was performed in the sub-second range with intervals that spanned 400–750 ms. As mentioned above, many empirical findings suggest that there is a perceptual dichotomy between sub- and supra-second timing (Wittmann, 2009) and it has been shown that different brain regions are engaged in sub and supra-second timing tasks (Lewis and Miall, 2003). Consequently, it is possible that memory biases are much smaller in the sub-second range where the interval encoding may be more accurate. In addition, the ratios of the reference durations used in that study were all less than the ratios used here (five for the 1/5 s group and two for the 2/4 s group). Since errors in time perception are known to grow with the duration of the intervals to be timed (Gibbon, 1977), we reason that errors in temporal memory may grow with the length of the intervals to be remembered and, consequently, memory biases in the bisection point may be larger for higher reference durations. Given our finding, it would be interesting in the future to administer the BiCaP task using reference durations in the sub-second range.

In addition to directly addressing the contribution of memory bias to the measurement of the bisection point, the results from the BiCaP task also afforded the opportunity to look at whether production and classification rely on common timing mechanisms, a question which has been elegantly addressed in prior work (Wing, 1980; Keele et al., 1985; Ivry and Hazeltine, 1995; Merchant et al., 2008, 2011). We were able to build on this body of work in several ways. First, whereas most previous studies have relied on comparisons across different experimental blocks, the BiCaP task involves comparisons among trial types within a single session. This approach may, therefore, better control for state effects, thereby decreasing the likelihood that subjects rely on short-term muscle memory to guide performance in production trials. It should be noted, however, that inter-mixing these trial types may affect the performance on each type. As it is known, for instance, that the order of reference duration presentation can affect the bisection location (Allan and Gerhardt, 2001), it is possible that the presence of a classification trial or a short reference production could affect, say, a midpoint production. It would be informative, therefore, to see how these results vary when trial-type blocks are used. Second, several of these studies have used rhythmic tapping tasks, in which the subject must produce periodic and repetitive movements, as a basis for comparison to classification. We chose a different approach as the motor movements themselves in tapping tasks contribute a large source of variability in the responses (Wing and Kristofferson, 1973; Wing, 1980). Third, while most previous human studies have focused on the sub-second range, we were interested in the seconds range of temporal perception. Using this method, we found that the bisection point derived from production and classification trials could not be distinguished (**Figure 2**, column 3). This lends support to the hypothesis that these distinct methods share at least partially overlapping timing mechanisms (Keele et al., 1985; Ivry and Hazeltine, 1995; Merchant et al., 2008).

We sought to address not only whether production and perception relied on similar timing mechanisms, but also what the nature of these mechanisms might be. By using two sets of referent probes (1/5 and 2/4 s) we were better able to address this question as the location of the bisection point derived from classification has been shown to vary with the ratio of the referents used (Wearden and Ferrara, 1996; Allan, 2002b). A meta-analysis showed that the bisection point will tend to be nearer the arithmetic mean for referent ratios exceeding four and be nearer the geometric mean for ratios lower than two (Kopec and Brody, 2010). Our reference sets spanned this range (ratio of 5 for the 1/5 s and ratio of 2 for 2/4 s group) so we were able to investigate this question. Interestingly, the raw bisection points for classification and production clearly did not accord with this trend and, in fact, were not consistent with any known model (Allan, 2002b). The fact that our raw bisection points do not accord with that of Wearden and Ferrara (1996) and others may be due to the fact that they use interval durations that are approximately an order of magnitude smaller; as it is known that different timing mechanisms are engaged for sub- and supra-second timing (Buhusi and Meck, 2005) the memory bias may not be as large a factor in this temporal regime. However, when we looked at the location of the bisection points with respect to bias-corrected measures of the arithmetic (**Figure 4**, green lines) and geometric (**Figure 4**, blue lines) means, we found significant evidence that the production bisection point followed this trend (though the classification data was likely underpowered to address this question). It is important to note, however, that the presence of the 1 s reference duration, which may engage multiple timing mechanisms as mentioned above, may affect the comparison between the 1/5 and 2/4 s groups. It is also important to note that, in addition to the reference duration ratio, the bisection point location can also be affected by the probe spacing and the presence of feedback. Here, we chose a linear probe spacing which, compared to logarithmic spacing, has been shown to push the bisection point closer to the arithmetic mean in human subjects (Wearden and Ferrara, 1995; Kopec and Brody, 2010). Therefore, it is possible that the bisection points in this study, which tended to be higher than the arithmetic mean, would have been pushed lower by a non-linear probe spacing. In addition, it is likely that the presence of feedback (similar to that used in training) on some fraction of trials would have yielded more veridical timing.

One aspect of our data violates a long-held tenet of time perception—scalar timing. Specifically, scalar expectancy theory predicts that as the standard deviation in temporal estimation grows with the temporal magnitude to be estimated (Gibbon, 1977). Although scalar timing is commonly cited as a fundamental feature of time perception, there are many examples in which it is violated (Lejeune and Wearden, 2006; Wearden and Lejeune, 2008). Our data adds to these examples, as Weber’s law was violated for production trials in that the CVs of the production for the short, long, and midpoint intervals were significantly different in the 1/5 s group. A potentially trivial explanation for this result is that the subjects were trained much more extensively on the referent intervals than on the midpoint intervals (which they were required to produce *de novo*, without any feedback, during testing). This does not explain why our results were consistent with Weber’s law in the 2/4 s group, however. Another explanation, alluded to above, is that the 1 s reference duration engages another timing mechanism from that engaged by the reference durations in the supra-second range. Interestingly, we did find evidence that the CV is inversely related to the bisection point on production trials in the 1/5 s group (slope = -8.53, *p* = 0.0042, *R*^2^ = 0.6627), as predicted by some theoretical accounts of timing (Balci et al., 2011; Namboodiri et al., 2014). This relationship was not significant for classification trials or for the 2/4 s group.

In sum, we have shown evidence that memory bias can account for variation in the location of the bisection point for both classification and production. In addition, by interleaving these trial types, we were able to simultaneously measure the classification and production bisection points and found that they were not significantly different, suggesting that these tasks rely on at least partially overlapping timing mechanisms. Using bias-corrected measures we were able to recapitulate some previously reported effects of the referent ratio on the bisection point, which non-corrected measures could not explain. These results suggest that it is important to consider memory bias in timing and design future experiments to measure its effects.

## Author Contributions

JL, VN, and MS conceived of the study. JL designed the code for the data collection. VN and JL performed the analyses. JL wrote the manuscript with help from VN and MS. JL and VN contributed equally to this work. The listing of first author was determined by a coin flip.

## Conflict of Interest Statement

The authors declare that the research was conducted in the absence of any commercial or financial relationships that could be construed as a potential conflict of interest.
